# Australia for the Sons of Professional Men

**Published:** 1908-04-25

**Authors:** Richard Arthur

**Affiliations:** President, Immigration League of Australasia.


### Editor's Letter-Box.

### AUSTRALIA FOR THE SONS OF PROFESSIONAL MEN.

Sir,—It is a good many years since I resided in Great Britain, but even then the problem of "what to do with our boys" pressed heavily upon the parents of the professional classes. The Army and Navy were closed to those who had no private income, the clerical, legal, medical, and other professions were overstocked, and the openings in commerce were only for those who had money or influential connections. And everything that I can learn points to an intensification of these conditions at the present day. The position, therefore, of the man who has sons about to leave school must be a very difficult one, when he is face to face with the question of how to give them a satisfactory start in life. It is in the hope of being able to do something to answer this question which prompts me to beg for some space in your columns.

For years many of the sons of professional men have emigrated to the United States or Canada. I come forward with the claim that Australia and New Zealand can offer opportunities superior to either of these two countries to industrious and persevering lads, who are prepared to accept rough and strenuous conditions for the sake of obtaining a competence in later life. I may say at once that there are no openings in our large towns. All the city occupations are filled to overflowing. But the Australian Bush is calling for thousands of willing hearts and hands to subdue it and make it fruitful.

The wealth of Australia is immense, and this is mostly due to the production of wool, wheat, sugar, butter, live-stock, and fruit—commodities for which there is a great and growing demand at prices that are likely to be maintained or increased. Life on the land therefore promises to him who approaches it in a spirit both scientific and practical, the opportunity, not only for a healthy and natural existence, but the possibility of an independence. The glorious climate of Australia is all in its favour. Here there is no long rigorous winter. A man can work and stock can graze in the fields all the year round. Much has been said about the Australian droughts, but it is not generally known that there are wide districts where drought is practically unheard of. And the conservation of fodder, and the great irrigation schemes which are being carried out, will henceforth rob the occasional droughts of much of their terror. In New South Wales there has been only one total failure of the wheat crops in 17 years. I do not for a moment suggest that lads should be sent out here with some money to take up land for themselves straight away. This would simply spell disaster. What I propose is that the lad who has decided to make Australia his home should adopt one of three courses.

1. That he should enter as a student at one of the Government Agricultural Colleges, where, for about £30 a year, he will receive a thorough training in scientific and practical farming. The sum quoted includes board and lodging, both of which I can guarantee to be excellent—quite as good, if not better than that provided at any English public schools. At the end of his two years' course there he could take up land for himself, or spend another year in gaining experience with a practical farmer before launching out for himself.

2. He could be placed, as far as New South Wales is concerned, with a farmer holding high qualifications as a teacher of agriculture, who for £12 a year and his labour would give him a training almost as good as that he could obtain at a college. I am again prepared to guarantee that any lads entrusted to this man would be properly looked after and instructed.

3. The third alternative is that he could go to a Government farm for three months and receive, free of cost, instruction in milking, ploughing, and the care of horses, pigs, etc. At the end of this time his services would be of sufficient value to enable him to be placed with a respectable farmer, who would pay him about 10s. a week and his board, and would increase his wages gradually to 15s. or 20s. After an apprenticeship of this kind for a year or two he would be fitted to start on his own account.

These three avenues to the land are open to all, and there exists a voluntary organisation here, the Immigration League of Australasia, whose chief object is to welcome newcomers, and afford them advice and assistance. The executive committee are prepared to give information to all who write to them, to arrange for the placing of lads at agricultural colleges or farms, to act generally "in loco parentis" towards them, and to see that they are not taken advantage of in any way. The League numbers among its members some of the leading men of Australia, and their desire is to help in the development of the continent, and retain the British emigrant under the flag of Empire.

We say, send your sons to us, and we will ensure them proper treatment and a fair start. We do not want ne'er-do-wells or failures. Experience proves that such do no better here than in the Old Country. But steady, industrious lads are bound to succeed here, and in many cases their parents might be induced to follow them out here, and prolong their lives in this incomparable climate. We invite parents to communicate with us at Bull's Chambers, Moore Street, Sydney, Australia, and we will give them full information, and send books and maps about Australia and New Zealand, and prospectuses of agricultural colleges and schools of mines. Some months' notice is needed to enable a lad to be entered at an agricultural college. Yours, etc.,

Richard Arthur, M.D.,
President, Immigration League of Australasia.

